# Effect of α^+^-thalassaemia on episodes of fever due to malaria and other causes: a community-based cohort study in Tanzania

**DOI:** 10.1186/1475-2875-10-280

**Published:** 2011-09-22

**Authors:** Jacobien Veenemans, Esther JS Jansen, Amrish Y Baidjoe, Erasto V Mbugi, Ayşe Y Demir, Rob J Kraaijenhagen, Huub FJ Savelkoul, Hans Verhoef

**Affiliations:** 1 Wageningen University, Cell Biology and Immunology Group, Wageningen, The Netherlands; 2 Muhimbili University of Health and Allied Sciences, Dar es Salaam, Tanzania, Tanzania; 3 Meander Medical Centre, Laboratory for Clinical Chemistry and Haematology, Amersfoort, The Netherlands; 4 London School of Hygiene and Tropical Medicine, London, UK; 5 Laboratory for Microbiology and Infection Control, Amphia Hospital, Breda, The Netherlands

## Abstract

**Background:**

It is controversial to what degree α^+^-thalassaemia protects against episodes of uncomplicated malaria and febrile disease due to infections other than *Plasmodium*.

**Methods:**

In Tanzania, in children aged 6-60 months and height-for-age z-score < -1.5 SD (n = 612), rates of fevers due to malaria and other causes were compared between those with heterozygous or homozygotes α^+^-thalassaemia and those with a normal genotype, using Cox regression models that accounted for multiple events per child.

**Results:**

The overall incidence of malaria was 3.0/child-year (1, 572/526 child-years); no differences were found in malaria rates between genotypes (hazard ratios, 95% CI: 0.93, 0.82-1.06 and 0.91, 0.73-1.14 for heterozygotes and homozygotes respectively, adjusted for baseline factors that were predictive for outcome). However, this association strongly depended on age: among children aged 6-17 months, those with α^+^-thalassaemia experienced episodes more frequently than those with a normal genotype (1.30, 1.02-1.65 and 1.15, 0.80-1.65 for heterozygotes and homozygotes respectively), whereas among their peers aged 18-60 months, α^+^-thalassaemia protected against malaria (0.80, 0.68-0.95 and 0.78, 0.60-1.03; p-value for interaction 0.001 and 0.10 for hetero- and homozygotes respectively). No effect was observed on non-malarial febrile episodes.

**Conclusions:**

In this population, the association between α^+^-thalassaemia and malaria depends on age. Our data suggest that protection by α^+^-thalassaemia is conferred by more efficient acquisition of malaria-specific immunity.

## Background

Alpha^+^-thalassaemia is a common genetic trait in malaria-endemic areas in sub-Saharan Africa, Asia and Melanesia, and may protect against the decline in haemoglobin concentration that is associated with asymptomatic *Plasmodium *infection [1].

Case-control studies [2-4] and two cohort studies [5,6] have consistently shown that α^+^-thalassaemia is associated with reduced risks of severe malaria. Reports on its effect on uncomplicated malaria are inconsistent. A study in Vanuatu showed that, among children aged < 5 years, homozygous (but not heterozygous) α^+^-thalassaemia was associated with an increased incidence of uncomplicated malaria due to both *Plasmodium vivax *and *Plasmodium falciparum *(incidence ratio, 95% CI: 2.2, 1.3-2.7 and 1.6, 1.0-2.6, respectively); paradoxically, among children aged 5-9 years, there was no evidence of such an association [7]. Subsequent studies in Africa found no effect [6,8,9] or protection [10] by α^+^-thalassaemia against uncomplicated malaria due to *P. falciparum*.

In a prospective case-control study in Papua New Guinean children, α^+^-thalassaemia was also strongly protective against hospitalization for disease episodes caused by infections other than malaria [2].

In this study among preschool children, it was aimed to assess effects of α^+^-thalassaemia on rates of fevers due to malaria and fever due to other causes, and evaluated to what extent these effects depended on age.

## Methods

### Study area and population

The study was conducted in February 2008-March 2009 in Handeni District, north-eastern Tanzania. The population is a mixture of many different tribes, residing in scattered hamlets, and mainly comprises poor farmer families engaged in subsistence farming. Transmission of malaria (> 95% due to *P. falciparum*) is intense and perennial, with entomological inoculation rates of 35-400 infectious bites per person-year [11]. Access to primary health care is limited. Artemether-lumefantrine was the first-line treatment for uncomplicated malaria, and available for free only in public care facilities, but not in local shops; accordingly, home treatment with efficacious anti-malarial drugs probably occurred only sporadically.

### Study design

This prospective cohort study was part of a double-blind randomized trial aimed at measuring the effects of preventive supplementation with zinc and other multi-nutrients on the incidence of malaria. Details about study design will be reported elsewhere. During the follow-up period, children received daily supplements under supervision. Parents were encouraged to bring their children to the research dispensary for fever or other signs of illness.

### Ethical approval

Ethical approval was provided by the review committees in The Netherlands and Tanzania. Individual written consent was obtained from parents.

### Recruitment

Before recruitment (February-August 2008), a census was conducted and all resident children aged < 60 months were registered. Parents and guardians were invited to bring their children for screening. A medical examination was performed by a clinical officer, anthropometric indices were calculated as the average of two recordings taken at consecutive days, and venous blood samples were collected. An aliquot of blood was centrifuged immediately after collection, and a 90 μL red cell pellet including the buffy coat was mixed with 90 μL phosphate-buffered saline and 180 μL of a DNA stabilizing buffer (AS1; Qiagen, Hilden, Germany) and stored at 4°C for subsequent genotyping. Plasma was stored in liquid nitrogen. A second aliquot of whole blood was examined by haematology analyzer (Sysmex KX21, Kobe, Japan) the same day.

Children were eligible for randomization when aged 6-59 months, and with height-for-age z-score < -1.5 SD. Children with weight-for-age z-score < -3 SD, haemoglobin concentration < 70 g/L, signs of severe or chronic disease, those unlikely to comply with interventions, or whose parents/guardians refused consent were excluded. Eligible children were randomly allocated to receive daily supplements with zinc alone, zinc in combination with other micronutrients, micronutrients alone, or placebo. Block randomization was used within six strata defined by *Plasmodium *infection and age class (6-17 months, 18-35 months and 36-60 months). Children with *Plasmodium *infection at baseline were treated with artemether-lumefantrine (Novartis Pharma, Basel, Switzerland).

### Follow-up

A clinical officer was on 24-h duty at the research clinic. For any child reporting with fever (axillary temperature ≥ 37.5°C) or a history of fever according to the guardian, a finger-prick blood sample was collected to detect the presence of malaria parasites. For all children with a positive dipstick we measured whole blood concentrations of haemoglobin and C-reactive protein. Children were treated free of charge, and referred to the district hospital when indicated.

### Laboratory procedures

In samples collected at baseline and from sick children, the presence of parasite-specific lactate dehydrogenase (*P. falciparum *and other *Plasmodium *species) was detected by rapid test (CareStart™, Access Bio, Monmouth Jct, USA). This test has a sensitivity of 96% for blood samples with > 50 parasites/μL as determined by microscopy [12]. Blood films were prepared using standard methods. For slides of sick children, parasites were counted against at least 200 leukocytes, and parasite density was calculated assuming 8,000 leukocytes/μL. At least 500 leukocytes were counted before a slide was considered negative. When densities were very high, parasites were counted per 2,000 erythrocytes, with estimates of erythrocyte density based on the haemoglobin concentration measured at the time of the episode, using a linear model based on survey data to describe the relationship between haemoglobin concentration and erythrocyte count.

DNA was isolated from erythrocyte pellets (Qiagen isolation kit); the -α^3, 7 ^deletion type of α^+^-thalassaemia was determined by polymerase chain reaction [13]. This type of deletion is the most common form of α^+^-thalassaemia in Africa [14], with prevalence values often exceeding 50% in eastern Africa [1,10,14]; other types of thalassaemia are rare.

Whole-blood concentrations of haemoglobin and C-reactive protein were measured using point-of-care tests (HemoCue, Ängelholm, Sweden and QuikRead, Orion Diagnostica, Espoo, Finland, respectively). Plasma concentrations of C-reactive protein and ferritin for survey samples were measured in The Netherlands (Meander Medical Centre, Amersfoort) on a Beckman Coulter Unicel DxC880i system according to the manufacturer's instructions. Plasma concentrations of *P. falciparum-*specific histidine-rich protein-2 (HRP2) in samples collected during the first malaria episode were measured using a commercial enzyme-linked immunosorbent assay kit (Malaria Ag Celisa; Cellabs, Brookvale NSW, Australia). This protein is released into the plasma at schizont rupture, and its plasma concentration may more accurately represent total body parasite biomass, because it also reflects the presence of sequestered parasites that remain undetected with conventional microscopy [15].

### Statistical analysis

Analyses were performed using SPSS (v15·0 for Windows, SPSS, Chicago, IL), CIA (v2.1.2) [16] and STATA (v11; College Station, Tx). Anthropometric indices were calculated using Epi Info software (version 3.3.2) [17].

The primary outcome, an episode of malaria, was pre-defined as a guardian-reported history of fever accompanied by either an axillary temperature ≥ 37.5°C or inflammation (whole blood C-reactive protein concentration ≥ 8 g/L), plus a positive result for the pLDH rapid dipstick test. Elevated C-reactive protein concentrations are probably indicative of recent malaria episodes in currently afebrile individuals with parasitaemia [18,19], and are associated with the severity of malaria (Veenemans and Verhoef, unpublished results). In the primary analysis, a parasite density threshold was not used to define malaria [20,21] because this can lead to biased estimates of effects when the interventions act by reducing (or increasing) parasite density [22,23], and density estimates can vary greatly within short time spans, ideally requiring leucocyte counts to be determined simultaneously [24,25]. To increase specificity of malaria case definitions, *Plasmodium*-infected participants were treated at baseline to clear parasitaemia before the start of surveillance. Episodes with parasitaemia thresholds of 5,000 and 10,000 parasites/μL were considered as secondary outcomes. Children were presumed to be protected against new malaria infections for 14 days after treatment with artemether-lumefantrine. Recurrent symptoms during this period were assumed to be part of the initial episode and were not counted as separate episodes. Additional outcomes were hospital admissions or death due to infection-related causes (combined end-point), non-malarial febrile episodes (defined as any episode of reported fever that did not classify as malaria, regardless of the presence or absence of parasitaemia, and separated by at least two days), and the severity of malaria episodes as indicated by parasite density, haemoglobin concentrations, concentrations of whole blood C-reactive protein and plasma HRP2.

In the primary analysis, group rates were compared using Cox regression with robust estimates of the standard error to account for multiple episodes within children. It was explored to what extent adjustment for baseline factors that were prognostic for malaria (age class, mosquito net use, *Plasmodium *infection, distance between homestead and clinic, and height-for-age z-score and micronutrient intervention) influenced the estimates. Because it was anticipated that the effect of α^+^-thalassemia would depend on age, a stratified analysis was conducted within each of the three age classes used for randomization, and interaction was assessed using a Cox regression model. It was also explored whether there was evidence of an interaction between α^+^-thalassaemia and the intervention by including interaction terms in the Cox regression model. Incidence and incidence ratios were calculated based on time to first episodes, and Cox regression models were used to obtain adjusted hazard ratios.

Associations between α^+^-thalassaemia and continuous variables (log-transformed as appropriate to obtain normal distributions) were assessed by ANOVA and multiple linear regression.

## Results

**Figure 1:**
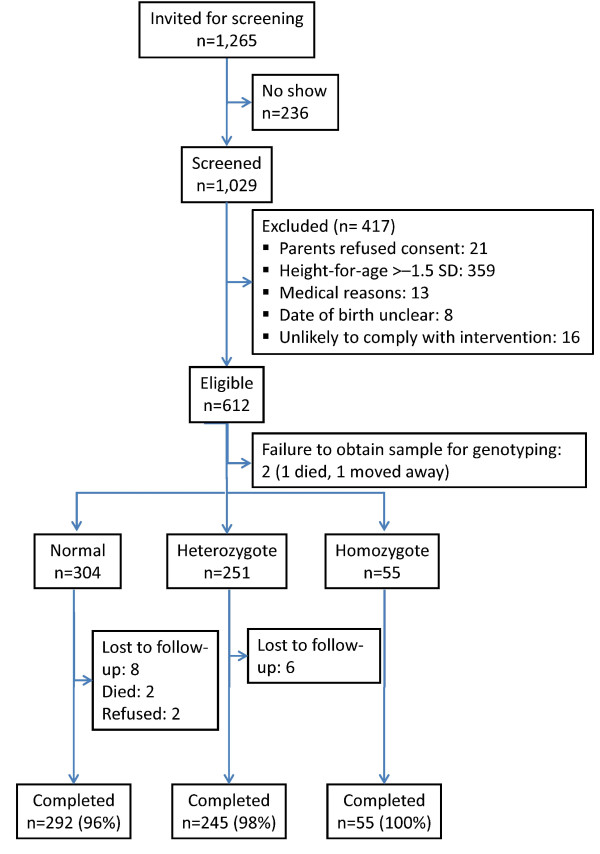
**Study profile**.

Of 1,029 children screened, 612 children were enrolled (Figure 1). During follow-up, 20 of 612 (3%) children were lost (three died; two were withdrawn by parents; 15 moved away).

Of 612 children included for follow-up, 304 (50%) had a normal genotype, 41% (251) were heterozygous, 9% (55) were homozygous; genotyping could not be performed for 2 children. *Plasmodium *infection was detected in 265 children (43%), virtually all due to *P. falciparum *(261), with similar prevalence values among genotypes. Homozygotes had lower haemoglobin concentrations and height-for-age z-scores, seemed slightly younger, appeared to have a higher prevalence of iron deficiency, and used mosquito nets less frequently than those with a normal genotype. They also less frequently received zinc supplements, with or without multi-nutrients (Table 1). Children with heterozygous and normal genotype were similar in factors prognostic for malaria. Haemoglobin concentrations were lower among children with α^+^-thalassaemia.

**Table 1:** Baseline characteristics of study participants and distribution of malaria prognostic factors, by genotype

	Normal (αα/αα)	Heterozygote (-α/αα)	p	Homozygote (-α/-α)	p
n	304 (50%)	251 (41%)		55 (9%)	
Sex, M/F [n/n]	50%/50% [151/153]	46%/54% [116/135]	0.44	54%/46% [30/25]	0.56
Age, months	33.4 ± 15.9	32.0 ± 15.2	0.27	29.9 ± 16.0	0.12
Age class			0.65		0.08
6-17 months	21% 64	24% [61]		35% [19]	
18-35 months	36% [108]	35% [87]		33% [18]	
36-59 months	41% [132]	41% [103]		42% [18]	
*Plasmodium *infection *	44% [134]	43% [107]	0.80	42% [23]	0.77
Anaemia ¶	62% [189]	71% [178]	0.03	87% [48]	< 0.001
Haemoglobin concentrations, g/L	104.7 ± 12.5	102.2 ± 12.4	0.02	94.9 ± 12.8	< 0.001
Without *Plasmodium *infection	106.7 ± 12.6	104.8 ± 11.6	0.14	95.3 ± 13.9	< 0.001
With *Plasmodium *infection	102.0 ± 11.8	98.8 ± 12.5	0.04	94.3 ± 11.3	0.005
Inflammation †	32% [99]	33% [82]	0.99	38% [21]	0.62
Mosquito net use ††	34% [101]	33% [82]	0.92	18% [10]	0.03
Height-for-age z-score	-2.38 ± 0.72	-2.43 ± 0.64	0.28	-2.63 ± 0.75	0.01
Distance from homestead to dispensary, km **	3.56 ± 2.21	3.65 ± 2.28	0.62	3.36 ± 1.84	0.54
Intervention					
Placebo	26% [78]	22% [54]		38% [21]	
Zinc	25% [76]	27% [67]		18% [10]	
Multi-nutrients without zinc	23% [71]	27% [68]		27% [15]	
Multi-nutrients with zinc	26% [79]	25% [62]		16% [9]	
Iron deficiency ∥					
All children	15% [46]	20% [49]	0.17	26% [14]	0.08
Without inflammation [n/n] §	22% [45/205]	25% [41/167]	0.80	35% [12/34]	0.13

Mean ± SD, % [n] or median (25- and 75-percentiles) unless indicated otherwise. P-values for differences relative to the reference group of children with normal genotype were obtained by Pearson Chi-Square test (age class), Fischer's Exact test (anaemia, *Plasmodium *infection, inflammation, mosquito net use, iron deficiency), or Students t-test (continuous variables). Because interventions were randomly allocated, no p-values are provided for intervention groups.

¶ Haemoglobin concentration < 110 g/L

* As indicated by a positive result for pLDH-based dipstick test (see text).

† Plasma C-reactive protein concentration ≥ 8 mg/L

†† Data missing for 11 children.

** Measured as the crow flies, based on global positioning data.

∥ Plasma ferritin concentration < 12 μg/L (6 missing values).

§ Restricted to children plasma C-reactive protein concentration ≥ 8 mg/L

Of 2,462 episodes of reported fever, 1,618 had a positive dipstick test result; 1,572 malaria episodes were recorded in 526 child-years of observation. The incidence of malaria was 3.0/child-year; 507 children (83%) experienced at least one malaria episode, recurrent episodes occurred in 395 (63%) of children. The remaining 890 febrile episodes were classified as not being due to malaria; of these, 27 (3%) were associated with a positive result for a malaria dipstick test, but in these cases, fever could not be confirmed and plasma C-reactive protein concentration was < 8 mg/L. The incidence of non-malarial fever cases was 1.69/child-year. Hospital referral for infection-related causes occurred in 68 cases, of which 30 were malaria (19 cases with haemoglobin concentration < 60 g/L, all except one without signs of heart failure or respiratory distress). Malaria incidence decreased with age class (3.73, 3.48 and 2.19 episodes/child-year for age classes 6-17, 18-35 and 36-59 months respectively; p < < 0.001); the corresponding incidence estimates for episodes with haemoglobin concentration < 80 g/L were 0.82, 0.33 and 0.09 episodes/child-year, respectively).

**Figure 2:**
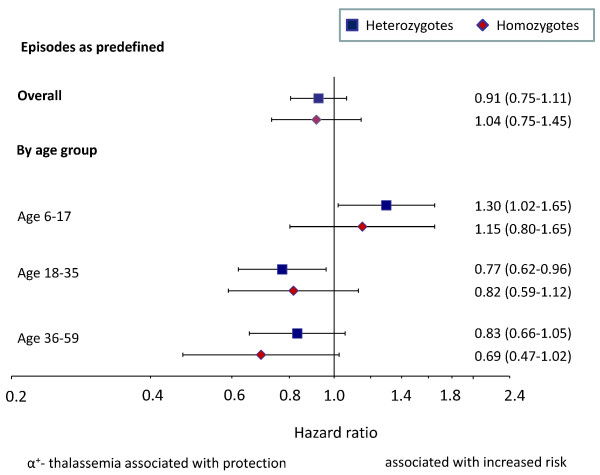
**Association between α^+^-thalassaemia and malaria rates, overall and by age class**. Episodes as predefined (see text). Line bars and values between brackets indicate 95% CIs. Estimates adjusted for baseline *Plasmodium *infection status, distance between homestead and clinic (continuous variable), height-for-age z-score (continuous variable), mosquito net use and experimental intervention.; corresponding p-values for the difference in hazard ratios between children < 18 months and ≥ 18 months: 0.006 and 0.18, for hetero-and homozygotes respectively.

**Figure 3:**
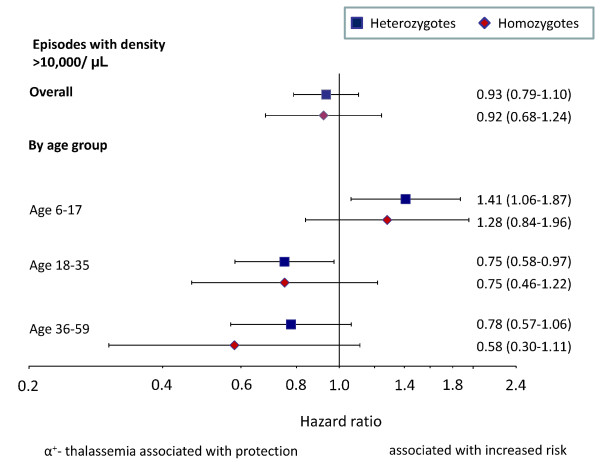
**Association between α^+^-thalassaemia and malaria rates, overall and by age class (episodes with densities > 10,000/μL)**. Malaria episodes as predefined, but with densities of asexual parasites > 10,000/μL. Estimates were adjusted as described in Figure 2; p-values for the difference in hazard ratios between children < 18 months and ≥ 18 months: 0.002 and 0.07, for hetero-and homozygotes respectively.

Rates of malaria were similar among genotypes (Table 2, Figure 2), whether analysed with or without adjustment for mosquito net use, *Plasmodium *infection at baseline, distance to the research clinic, stunting and experimental intervention. Stratification by age-class, however, showed that heterozygotes had increased malaria rates when aged < 18 months (hazard ratio: 1.30, 1.02-1.65), whereas they were protected against malaria when aged ≥ 18 months (Figure 2; hazard ratio: 0.80, 0.68-0.95; p-value for interaction: 0.001). A similar pattern occurred in homozygotes, even though estimates were less precise due to the smaller number of cases (hazard ratio: 0.78, 0.60-1.03; p-value for interaction: 0.10). Restriction of the analysis to malaria cases with parasite densities above 5,000/μL, 10,000/μL or 100,000/μL resulted in similar overall estimates (Table 2), and similar age-dependent patterns with and without adjustment for baseline factors prognostic for malaria (data shown for episodes with > 10,000 parasites/μL in Figure 3; p-values for interaction: 0.001 and 0.05, for heterozygotes and homozygotes respectively). Similar effect estimates were also obtained when taking account potential interaction between the intervention and α^+^-thalassaemia, and there was no evidence that the magnitude of the effects of α^+^-thalassaemia on malaria rates were influenced by the intervention (p = 0.94 and 0.95 for interaction with heterozygotes and homozygotes, respectively).

**Table 2:** Rates of uncomplicated malaria, non-malarial febrile episodes and severe events (hospital admission or death), by genotype

**Event**	**Normal** **(αα/αα)**		**Heterozygotes** **(-α/αα)**		**Homozygotes** **(-α/-α)**	
			
**All episodes of malaria**						
Incidence (n/child-years)	3.10	(812/262)	2.89	(622/215)	2.83	(136/48)
Hazard ratio, crude	1.00	Reference	0.93	[0.80-1.06]	0.92	[0.73-1.14]
Hazard ratio, adjusted †	1.00	Reference	0.93	[0.82-1.06]	0.91	[0.73-1.14]
Episodes with parasitaemia > 5,000/μL (1,249)	1.00	Reference	0.94	[0.81-1.10]	0.89	[0.67-1.17]
Episodes with parasitaemia > 10,000/μL (1,119)	1.00	Reference	0.95	[0.81-1.11]	0.91	[0.68-1.22]
Episodes with parasitaemia > 100,000/μL (263)	1.00	Reference	0.94	[0.69-1.29]	0.98	[0.57-1.69]
Episodes with haemoglobin concentration < 80 g/L (178)	1.00	Reference	1.25	[0.86-1.84]	2.65	[1.71-4.10]
			
**1^st ^episodes of malaria**						
Incidence (n/child-years)	3.08	(257/84)	2.66	(199/75)	3.44	(49/14)
Incidence rate ratio	1.00	Reference	0.86	[0.71-1.04]	1.12	[0.81-1.52]
Hazard ratio, crude	1.00	Reference	0.90	[0.75-1.09]	1.10	[0.81-1.49]
Hazard ratio, adjusted †	1.00	Reference	0.89	[0.74-1.08]	1.06	[[0.78-1.44]
			
**All episodes of non-malarial fever**						
Incidence (n/child-years)	1.64	(431/262)	1.72	(370/215)	1.82	(87/48)
Hazard ratio, crude	1.00	Reference	1.04	[0.85-1.28]	1.10	[0.82-1.49]
Hazard ratio, adjusted ¶	1.00	Reference	1.00	[0.84-1.19]	0.98	[0.73-1.36]
			
**All hospital admissions or deaths §**						
Incidence (n/child-years)	0.15	(39/262)	0.10	(21/215)	0.19	(9/48)
Hazard ratio, crude	1.00	Reference	0.66	[0.38-1.14]	1.26	[0.56-2.89]
Hazard ratio, adjusted ¶	1.00	Reference	0.57	[0.33-0.98]	0.89	[0.36-2.24]

Values between brackets indicate (cases), (cases/child-year) or [95% CIs].

† Adjusted for experimental intervention (indicated by dummies), mosquito net use, distance between homestead and research clinic, height-for-age z-score and *Plasmodium *infection at baseline. There was no evidence of an interaction between genotype and experimental intervention.

¶ Adjusted for age class, experimental intervention (dummies), distance between homestead and research clinic, height-for-age z-score and *Plasmodium *infection at baseline.

§ Hospital admissions or deaths (of which two occurred outside hospital) for infection-related causes, excluding events due to trauma, poisoning or burns (one case occurred in a child for whom the genotype could not be determined).

The rate of episodes with haemoglobin concentrations < 80 g/L was highest among homozygotes and lowest among children with a normal genotype, regardless of age.

**Figure 4:**
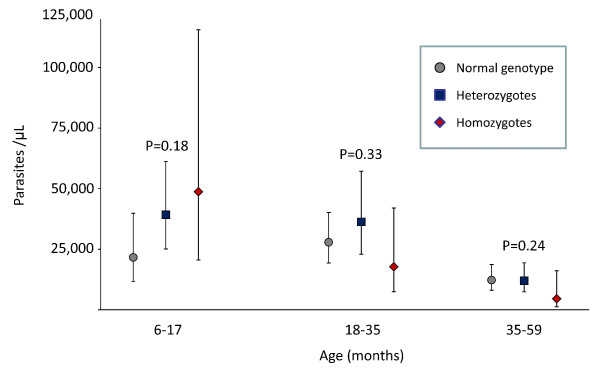
**Association between α^+^-thalassaemia and parasite density during first episode of malaria, by age class**. Markers and line bars indicate geometric means and 95% CIs, respectively; p-values for differences between genotypes within age classes obtained by ANOVA).

When genotype was not taken into account, parasite densities during the first malaria episode declined with age (p < < 0.001; Figure 4). In children aged below 18 months, α^+^-thalassaemia seemed associated with increased parasite densities (Figure 3), although the statistical evidence for such an association was weak (p = 0.18). For homozygotes, the association between genotype and density seemed to depend on age (p = 0.04), while for heterozygotes there was no such evidence (p = 0.78). α^+^-thalassaemia was not found to be associated with parasite density in older age groups (Figure 4). Other indicators of severity (plasma concentrations of C-reactive protein or HRP2) were comparable between genotypes, whether analyzed for all age classes combined or separately (data not shown).

The declines in average haemoglobin concentrations between baseline and the first malaria episode were only minor and not substantially different in hetero- or homozygotes (-4.5 g/L and -2.1 g/L) from values observed in children with a normal genotype (-3.0 g/L).

Lastly, the rate of non-malaria febrile episodes was similar between genotypes (Table 2); stratification by age did not indicate differences in age-specific effects.

## Discussion

In this cohort, α^+^-thalassaemia was associated with increased rates of malaria in children aged < 18 months, but with protection against malaria in older children. There was no evidence that α^+^-thalassaemia was associated with the severity of malaria episodes as measured by haemoglobin concentrations and other indicators.

Although all *Plasmodium*-infected children were treated at baseline, the specificity of our case definition may have gradually decreased with time, as more children became asymptomatically infected. Similar results were obtained, however, when restricting the analysis to cases with parasite densities > 10,000/μL or > 100,000/μL. Because these case definitions are more specific for detecting true malaria cases, it is unlikely that a low specificity affected the validity of the conclusions drawn in this report.

Because effect estimates were adjusted for factors associated with malaria risk (including distance to the research facility and intervention), it is unlikely that a difference in external factors (such as intervention, exposure to infection or health seeking behaviour) biased the estimates and would be responsible for the increased risk associated with α^+^-thalassaemia in the youngest children. It remains unclear however, what mechanism could underlie the increased incidence in the youngest children. It has been put forward that *Plasmodium *parasites preferentially invade reticulocytes [26,27]. Thus reticulocytosis, induced by thalassaemia-associated ineffective erythropoiesis, would favour proliferation of *Plasmodium *parasites [28]. However, there was no strong support that thalassaemia was associated with increased parasite densities in young children, and neither did any previous study. In addition, a recent study found no evidence that reticulocyte counts were increased in individuals with α^+^-thalassaemia [29].

Others hypothesized that increased parasite-induced surface expression of neo-antigens on thalassaemic erythrocytes results in enhanced binding of IgG antibody and more rapid clearance of parasitized erythrocytes in the spleen [30]. Clearance in the spleen may be further enhanced by a reduced erythrocyte deformability of thalassaemic cells [31]. In children aged 6-18 months, in whom protection by maternal antibodies has waned, but acquired immunity is still low and parasite replication only partly restrained by an effective circulating antibody repertoire, such increased antigen presentation in the spleen may result in a more rapid development of symptoms, and at the same time a more efficient and more rapid acquisition of protective immunity. Further evidence to support this theory is, however, lacking.

Nevertheless, the findings reported here contribute to existing epidemiological evidence that predisposition to malaria due to *P. falciparum *in both heterozygotes and homozygotes for α^+^-thalassaemia early in life may result in protection against severe malaria, and (at older age) uncomplicated malaria due to the same species. In Vanuatu, the incidence of malaria due to *P*. *falciparum *and *P. vivax *was increased in children aged < 5 years with homozygous α^+^-thalassaemia relative to children with normal genotype [7], but the study found no evidence of protection among either hetero- or homozygotes in children aged 5-9 years. Contrary to our findings, the incidence in heterozygotes and children with normal genotype were similar, regardless of whether the analysis was stratified by age or not. The estimates in the current report are more precise, however, due to the larger number of malaria cases in this study (622 and 812 in children with heterozygote and normal genotypes, respectively, versus 159 and 304 in Vanuatu). In an area adjacent that used in the current report, with similar levels of malaria endemicity, α^+^-thalassaemia was found to be associated with protection against malaria in children aged 6 months to 20 years [10]. This protection seemed more pronounced among children aged > 5 years, but the analysis was based on 50 episodes (41 among children aged < 5 years) and had insufficient precision to adequately assess age-specific effects in early life.

Because the intensity of malaria exposure determines how fast protective immunity is obtained, the age at which a difference in protective immunity between children with and without α^+^-thalassaemia becomes evident shall vary with transmission intensity. This may at least in part explain differences between studies; under conditions of intense transmission, such as in the present study, the difference attained would become evident earlier in life than in conditions of less intense transmission such as encountered in Vanuatu [7].

The finding that α^+^-thalassaemia is associated with an increased frequency of malaria in children aged 6-18 months may seem to contradict reports from hospital-based studies that α^+^-thalassaemia protects against severe malarial anaemia [4,6], which has the highest incidence in the same age range [32]. An increased fever rate may, however, not necessarily translate to an increased risk of severe malaria anaemia if the decline in haemoglobin during these attacks is halted or sufficiently slowed down to before reaching a critical level that leads to admission. A potential mechanism for such phenomenon has recently been proposed [33]. In thalassaemia, the total amount of haemoglobin is divided over erythrocytes that are disproportionately increased in numbers but reduced in size and haemoglobin content. Thus, an equal proportion of erythrocytes being destroyed by malaria parasites results in a smaller haemoglobin reduction in individuals with α^+^-thalassaemia than in their peers with normal genotype.

When analysing the decline in haemoglobin concentration between baseline and first malaria episode, we did not find evidence of such protection. It should be noted, however, that the average decline in haemoglobin concentration during malaria episodes was relatively small, and that very few (19) episodes occurred whereby haemoglobin concentrations dropped below 60 g/L. This is probably due to good access to treatment in our study, and haematological gains due to the nutrient interventions. By contrast, in a pilot survey in the same area in 2006, children with α^+^-thalassaemia were found to be protected against the decline in haemoglobin concentration associated with mild and asymptomatic infections [1]. At that time, there were no first-line health facilities in the area, and artemether-lumefantrine was not available through public facilities. Thus this setting was probably better comparable to the circumstances under which α^+^-thalassaemia has been providing a survival advantage in the past.

A likely explanation for the increased rates of episodes with haemoglobin concentrations < 80 g/L observed in heterozygotes and homozygotes (Table 2) is that children with these genotypes had lower initial haemoglobin concentrations before the onset of malaria episodes than children with normal genotype. Thus a reduction in haemoglobin concentration caused by malaria in heterozygotes and homozygotes will more readily result in haemoglobin concentrations dropping below a threshold of 80 g/L.

## Conclusions

In pre-school Tanzanian children living in an area of intense transmission, and with height-for-age z-score < -1.5 SD, effects of α^+^-thalassaemia on malaria rates were age-dependent: it was associated with increased rates in children aged < 18 months as opposed to decreased rates in older children. There was no evidence that α^+^-thalassaemia was associated with the severity of malaria episodes.

## Competing interests

The authors declare that they have no competing interests.

## Authors' contributions

JV was responsible for data collection and analysis, administration and drafted the manuscript; HV assisted in statistical analyses. EJSJ, EVM and AB assisted in data collection; RJK and AYD were responsible for laboratory analyses in The Netherlands. HV and HFJS were responsible for supervision. All authors participated in data interpretation and critical revision of the report for intellectual content; and provided final approval of the submitted version.
